# The safety of Ramadan Fasting following Percutaneous Coronary Intervention

**DOI:** 10.1186/s12872-020-01784-8

**Published:** 2020-11-19

**Authors:** Osama A. Amin, Ahmed Alaarag

**Affiliations:** 1 Department of Cardiology, Beni Suef University, Beni Suef, Egypt; 2 Department of Cardiology, Tanta University, Tanta, Egypt

**Keywords:** Ramadan Fasting, Percutaneous Coronary Intervention, Major adverse cardiovascular events

## Abstract

**Background:**

This work aimed to assess the safety of Ramadan Fasting following the Percutaneous Coronary Intervention.

**Methods:**

In our two centers’ Prospective Cohort Study, We included 303 patients who had successful Percutaneous Coronary Intervention before the first day of Ramadan. We advised the patients that recent Percutaneous Coronary Intervention could be a valid excuse for not fulfilling Ramadan Fasting. However, many patients intended to fast the following Ramadan, and we included them in the fasting Group I. We added the patients who decided not to fast the following Ramadan as a control Group II. We followed all the patients during Ramadan and for 6 months after Ramadan.

**Results:**

The demographic data of both groups and the complexity of the coronary anatomy showed no statistically significant difference. Group I (n = 153) showed a statistically significant difference in the incidence of Major Adverse Cardiac Events compared to Group II with a *P* value (0.005). The logistic multivariate regression analysis showed that the duration from index PCI till the start of RF, SYNTAX score > 22, and Complex procedure were independent predictors of Major Adverse Cardiac Events in the fasting Group I with {*P* = 0.001, OR (2.302), *P* = 0.026, OR (2.419), and *P* = 0.032 OR (1.952)}, respectively. Major Adverse Cardiac Events in Group I occurred mainly during Ramadan Fasting, with 19 patients having Major Adverse Cardiac Events during Ramadan and four patients during the remaining of the follow-up period. The Receiver Operating Characteristic curve analysis showed the decline of the incidence of Major Adverse Cardiac Events after 90 days from Percutaneous Coronary Intervention till the start of Ramadan Fasting with Sensitivity and specificity (90% and 65%), respectively.

**Conclusions:**

We suggest that low-risk patients with a normal systolic function who underwent Percutaneous Coronary Intervention may safely fast Ramadan. At the same time, Ramadan Fasting during the first 3 months following the Percutaneous Coronary Intervention may not be safe.

## Background

Ramadan Fasting (RF) is considered one of the pillars of Islam. RF includes refraining from any form of ingestion into the body from dawn until Sunset. RF is mandatory for all adult Muslims except for patients with medical disorders that exclude them from fasting. Although physicians always exempt patients with illnesses from this religious obligation, many continue with fasting for spiritual reasons, and they strictly organize their prescription timetable to outfit RF. The metabolic and clinical effects of RF have been previously studied systematically [1]. Most of the studies suggested that RF is safe in stable patients with Coronary Artery Disease (CAD) [2, 3].

RF reduced the proinflammatory markers significantly 4 weeks after Ramadan, denoting a potential long-term beneficial RF role on proinflammatory markers [4]. Most of the studies followed the patients for 4–12 weeks after RF. Hence, they recommended further studies with longer follow up [4–6]. Besides, RF modified light and food cycles, leading to a disruption of the circadian rhythm during and even after RF [7, 8].

Thrombogenicity, aggravated by dehydration, plays a critical role in favoring late stent thrombosis [9, 10]. Many cardiologists advise patients with recent Percutaneous Cardiac Intervention (PCI) to avoid fasting because of potential dehydration, which may increase the risk of stent thrombosis. Although there are no consensus guidelines, it looks sensible to advise patients with myocardial infarction, unstable angina (UA), or recent revascularization to avoid RF [2].

However, the safety of RF following PCI remains very debatable. Regarding us, there were no studies that followed patients fasting Ramadan after PCI (They were followed as subgroups with a small number in larger studies) [1–3] but without any clear recommendations to fast a few months after PCI. Muslims are present in many geographical regions with variable climates and daylight hours. So we need multicenter studies from different countries to evaluate a suitable period after PCI to allow RF. We tried to relatively share our limited experience to answer a question that is frequently asked by many patients after PCI (when am I allowed to fast Ramadan?). The clear answer, to our knowledge, is not yet available in the literature.

### Aim of the work

This work aimed to assess the safety of RF after PCI.

## Methods

In our two centers’ Prospective Cohort Study, We initially included 313 patients < 75 years who had successful PCI with complete revascularization with DES before the first day of Ramadan. We included 155 patients from Beni Suef University Hospital and 158 patients from Tanta University Hospital. The board of research and medical ethics of the Cardiology Department accepted the study protocol in February 2018. We obtained informed written consent from all the patients. We included the patients who had successful PCI with complete revascularization and excluded any patient with any of the following criteria:

1. The patients with any comorbidities preventing fasting: i.e., acute conditions like infections or chronic severe obstructive airway disease requiring frequent treatment, or decompensated hepatic disease.
2. Advanced renal impairment with Estimated Glomerular Filtration Rate less than 60 mL/min/1.73 m^2^, or Hyperkalemia (> 5.5 mmol/L), or Low blood pressure symptoms, or Hypovolemia, or Dehydration, or Cardiac Outflow Obstruction.
3. The patients with incomplete revascularization or persistent ischemic symptoms after PCI
4. The patients with impaired systolic function [Ejection Fraction (EF) < 50%].
5. The patients with Insulin Dependent Diabetes Mellitus (IDDM) or uncontrolled Diabetes Mellitus (DM) defined as HBA1c > 7%.

We advised the patients that recent PCI could be a valid excuse for not fulfilling the obligation of RF. However, many patients insisted and intended to fast the whole following Ramadan, and we included them in the fasting Group I. We added the patients who decided not to fast the following Ramadan as a control Group II. We informed the patients about breaking their fast once they experience any symptoms such as chest pain or shortness of breath.

Regarding the patients in the fasting group, we adjusted all the medications twice daily (at the two meals: Sahur and Iftar). We started to call the 313 patients fulfilling the inclusion criteria for the baseline assessment on the 9th of May 2018 (1 week before Ramadan), and we continued the enrollment process till the 15th of May 2018 (the day before the first day of Ramadan). Ramadan ended on the 14th of June 2018. We followed the patients until December 2018 (6 months after Ramadan). We scheduled two routine visits: 25–30 days after the beginning of Ramadan and 6 months after the end of Ramadan. If there was a need for hospital admission or clinic visit due to a change in symptoms, we performed the proper management according to the current guidelines.

We further excluded from our study:

1. The patients with any Major Adverse Cardiac Events (MACEs) before the first day of Ramadan. MACEs included hospitalization for Non-ST Elevation Myocardial Infarction (NSTEMI), UA, ST-Segment Elevation Myocardial Infarction (STEMI), death, stroke or Transient Ischemic Attack (TIA), hospitalization for other cardiovascular reasons. We considered the first event for each patient as a primary outcome.
2. The patients who did not attend any follow-up visit or did not comply with the evidence-based prescribed medications, especially the dual antiplatelet therapy.

**Fig. 1:**
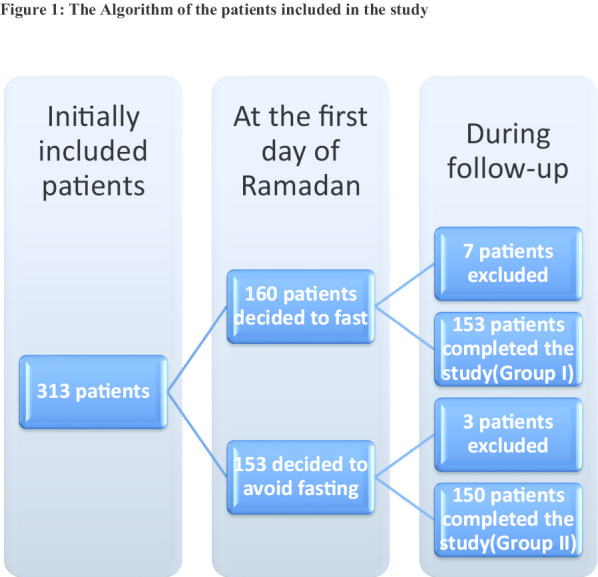
The algorithm of the patients included in the study

So the final sample size after the further exclusion was 303 patients, as shown in Fig. 1.

We subjected all the patients to the following at the baseline (within 1 week before Ramadan), the first follow up visit (25–30 days after fasting), and the second follow up visit (6 months after the end of Ramadan): history taking, demographic data, Clinical examination [to exclude the patients with evidence of any of the exclusion criteria and to assess the NEW YORK Heart Association functional class(NYHA)], routine laboratory test (to exclude patients with uncontrolled DM, renal impairment, or Hyperkalemia), Electrocardiogram (to monitor any changes from the baseline), and transthoracic echocardiography (using standard views to assess the Left Ventricular End-Diastolic Volume, Left Ventricular End-Systolic Volume, and the EF was calculated by the modified Simpson’s Method, following the guidelines of the European Association of Cardiovascular Imaging [11]. We registered the first MACE in any patient during the holy month Ramadan and 6 months after Ramadan as the primary outcome. MACEs included hospitalization for NSTEMI, UA, STEMI, stent thrombosis, death, stroke, or TIA, hospitalization for other cardiovascular reasons, or Target Lesion Revascularization (TLR).

### Statistical analysis

We used the Statistical Program for Social Science (SPSS) version 20.0. We used the Kolmogorov–Smirnov test for testing the normal distribution of continuous data, and our data were found to be normally distributed. We expressed Quantitative data as mean ± Standard Deviation (SD). We showed Qualitative data as frequency and percentage. We used the Chi-square (X2) test of significance to compare categorical variables and the Student *t* test for continuous variables between the two groups. We performed univariate and logistic multivariate regression analysis to study the multiple variables which may affect the occurrence of MACEs. We studied MACEs as a dependent variable with basal demographic characteristics and details of PCI procedures as independent covariates. We used the logistic multivariate regression analysis to adjust the different potential confounders (covariates), affecting the MACEs. We presented the odds ratio for univariate analysis and the adjusted odds ratio for the multivariate analysis with 95% confidence intervals. We used Receiver Operating Characteristic curve analysis (ROC) and the Area Under Curve (AUC) to predict the accuracy. We selected the best cutoff value for the number of days from index PCI till the start of RF at which there is a significant change in the incidence of MACEs. The limit of statistical significance was 0.05.

## Results

In our two centers' Prospective Cohort Study, we finally followed 303 patients after applying the exclusion criteria. One hundred fifty-three patients intended to fast the whole month despite having the religious permission to avoid fasting after PCI (Group I). The non-fasting control (Group II) included 150 patients, as shown in Fig. 1.

Ramadan, in our study (2018, 1439 Hijri), was 30 days. There were nearly the same daylight temperature, daylight humidity, and fasting hours in the two cities where we conducted the study. The mean daylight temperature during fasting hours was 32° and 34° in the city I and city II, respectively. The mean daylight humidity during fasting hours was 38% and 36% in city I and city II, respectively. The mean fasting hours during the month were 15:41 and 15:35 in city I and city II, respectively.

The basal demographic data of both groups showed no statistically significant difference, as shown in Table 1.

**Table 1:** The risk factors and demographic data in both groups

	Group I (n = 153)	Group II (n = 150)	*T* test	*P* value
Age				
Range	40–70	42–70	1.356	0.245
Mean ± SD	58.12 ± 7.28	57.09 ± 8.02		

	Group I	Group II	X^2^	*P* value
Gender				
Male				
N	105	106	0.149	0.700
%	68.6%	70.7%		
Female				
N	48	44		
%	31.4%	29.3%		
DM				
Yes				
N	27	29	0.143	0.705
%	17.6%	19.3%		
No				
N	126	121		
%	82.4%	80.7%		
HTN				
Yes				
N	40	32	0.9689	0.325
%	26.1%	21.3%		
No				
N	113	118		
%	73.9%	78.7%		
Current Smokers				
Yes				
N	42	38	0.175	0.676
%	27.5%	25.3%		
No				
N	111	112		
%	72.5%	74.7%		
Dyslipidemia				
Yes				
N	60	53	0.488	0.485
%	39.2%	35.3%		
No				
N	93	97		
%	60.8%	64.7%		

*SD* Standard Deviation, *DM* diabetes, *HTN* hypertension

*Significant *P* value

Also, there was no statistically significant difference between both groups regarding the P2Y12 inhibitors used and the indication for PCI. We found no significant difference between the two groups regarding the days from index PCI to the first day of the following Ramadan with *P* value = 0.522, as shown in Table 2.

**Table 2:** Days from index PCI to the first day of Ramadan, indication of PCI, and P2Y12 inhibitor used and total MACEs in both groups

	Group I (n = 153)	Group II (n = 150)	*T* test	*P* value
Days from index PCI to the first day of Ramadan				
Range	65 – 170	62 – 265	0.411	0.522
Mean ± SD	170.07 ± 64.21	165.43 ± 61.47		

	Group I	Group II	X^2^	*P* value
P2Y12 Inhibitor				
Clopidogrel				
N	97	101	0.518	0.472
%	63.4%	67.3%		
Ticagrelor				
N	56	49		
%	36.6%	32.7%		
Indication for PCI				
ACS				
N	19	18	0.012	0.911
%	12.4%	12.0%		
Elective				
N	134	132		
%	87.6%	88.0%		
MACEs				
Yes				
N	23	8	7.759	0.005*
%	15.0%	5.3%		
No				
N	130	142		
%	85.0%	94.7%		

*SD* Standard Deviation, *PCI* Percutaneous coronary intervention, *ACS* Acute Coronary Syndrome, *MACEs* Major Adverse Cardiac Events

*Significant *P* value

There was no significant difference in PCI details and the complexity of the coronary anatomy, as presented by the SYNTAX score between both groups, as shown in Table 3.

**Table 3:** Details of PCI procedures in both groups

	Group I (n = 153)	Group II (n = 150)	X^2^	*P* value
Patients with LM stents				
N	4	3	0.127	0.722
%	2.6%	2%		
Patients with SYNTAX score ≤ 22				
N	93	90	0.019	0.889
%	60.8%	60%		
Patients with SYNTAX score > 22				
N	60	69		
%	39.2%	40%		
Patients with bifurcation stents				
N	3	3	0.001	0.980
%	1.9%	2%		
Sirolimus eluting DES				
N	125	121	0.053	0.818
%	81.7%	80.7%		
Non Sirolimus eluting DES				
N	28	29		
%	18.3%	19.3%		
Patients with overlapped stents				
N	80	79	0.024	0.876
%	52.2%	52.6%		
Use of NC balloons for stent optimization				
N	90	90	0.043	0.835
%	58.8%	60%		

	Group I (n = 153)	Group II (n = 150)	*T* test	*P* value
Length of stents in each patient (mm)				
Range	12–133	15–132	0.829	0.373
Mean ± SD	45.29 ± 19.34	42.85 ± 18.16		
Amount of contrast used (ml)				
Range	100–350	90–350	− 1.345	0.180
Mean ± SD	151.91 ± 41.56	158.38 ± 56.28		

*LM* left main, *DES* drug eluted stent, *NC* non-compliant

**Fig. 2:**
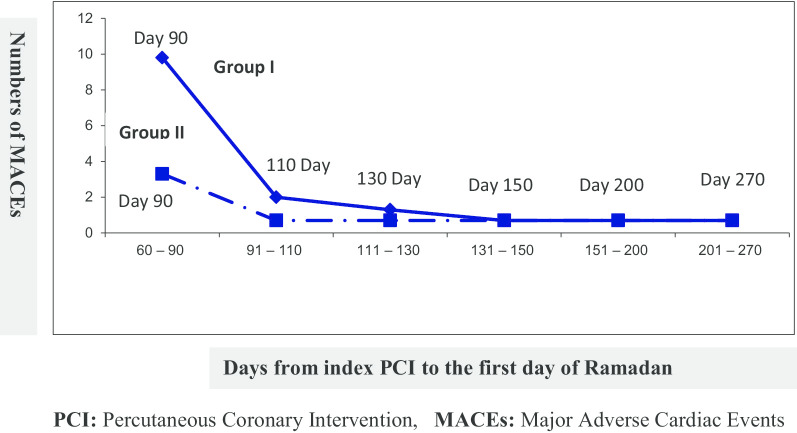
The relation between MACEs and Days from index PCI to the first day of Ramadan in both groups

Group I (n = 153) showed a statistically significant difference in the incidence of MACEs compared to Group II with a P-value (0.005), as shown in Table 2 and Fig. 2. Group I had 15% MACE (23 patients) compared to 5.3% (8 patients) in Group II. Also, the majority of MACEs in Group I occurred when the patients started RF ≤ 90 days from the index PCI (15 patients, 9.8%), while in Group II (n = 150), only (3 patients, 2%) had MACEs for the same follow up period (7 months). We also noticed that the difference in the incidence of MACEs between both groups decreased when the patients started RF > 90 days from index PCI. The incidence of MACEs was equal in both groups when the patients started RF ≥ 131 days from the index PCI. Most of the MACEs in patients who started RF ≤ 90 days after stent deployment were related to stent thrombosis), and most of them had SYNTAX score > 22, or complex PCI procedures, as shown in Fig. 2 and Tables 4 and 6.

**Table 4:** The relation between MACEs and the days from index PCI to the first day of RF in Group I

	The days from index PCI to the first day of RF Group I (n = 153)													
	60–90		91–110		111–130		131–150		151–200		201–270		Total	
	N	%	N	%	N	%	N	%	N	%	N	%	N	%
Hospitalization for Non-STEMI or UA	10	6.5	1	0.7	2	1.3	1	0.7	1	0.7	1	0.7	16	10.5
STEMI	4	2.6	0	0	0	0	0	0	0	0	0	0	4	2.6
Death	1	0.7	0	0	0	0	0	0	0	0	0	0	1	0.7
Stroke or TIA	0	0	0	0	0	0	0	0	0	0	0	0	0	0
Hospitalization for other cardiovascular reasons	0	0	2	1.3	0	0	0	0	0	0	0	0	2	1.3
Total patients with MACEs	15	9.8	3	2	2	1.3	1	0.7	1	0.7	1	0.7	23	15

*MACEs* Major Adverse Cardiac Events, *UA* unstable angina, *RF* Ramadan Fasting, *Non-STEMI* Non-ST-Segment Elevation Myocardial Infarction, *TIA* Transient Ischemic Attack, *STEMI* ST-segment Elevation Myocardial Infarction

In Group I, we admitted 16 patients with NSTEMI or UA; 62.5% of them (n = 10) underwent PCI ≤ 90 days before starting RF. All the patients who presented with (STEMI) (n = 4), deaths (n = 1), and stent thrombosis (n = 7) underwent the PCI ≤ 90 days before starting RF, as shown in Table 5. Also, we found that nine of the ten patients who needed (TLR) underwent the PCI ≤ 90 days before starting RF, as shown in Tables 4 and 5. We admitted two patients to the hospital, and both of them started RF > 90 days from the index PCI. However, we admitted one of them due to having gastrointestinal bleeding (major bleeding event). We admitted the second patient with atrial fibrillation and mesenteric vascular occlusion because of improper anticoagulation.

**Table 5:** Distribution and details of MACEs in Group I during the follow-up

	MACEs during RF		MACEs following RF		Total		Comments
	N	%	N	%	N	%	
Hospitalization for Non-STEMI or UA	14	9.2	2	1.3	16	10.5	Three patients of them had a definite stent thrombosis at coronary angiography (all of them occurred during RF)
STEMI	4	2.6	0	0	4	2.6	Three patients of them had a definite stent thrombosis at coronary angiography (all of them occurred during RF)
							One of those three patients had a cardiac arrest on the second day of admission
Death	1	0.7	0	0	1	0.7	The patient presented by sudden cardiac death with his ECG after resuscitation showing STEMI (probable stent thrombosis)
Stroke or TIA	0	0	0	0	0	0	
Hospitalization for other cardiovascular reasons	0	0	2	1.3	2	1.3	We admitted two cases to the cardiology department with symptomatic hypotension: One with GIT bleeding and the other one with ischemic mesenteric embolic vascular occlusion
Total patients with MACEs	19	12.4	4	2.6	23	15	Seven cases of stent thrombosis (all of them occurred during RF), and ten cases of TLR (Nine of them occurred during RF)

*MACEs* Major Adverse Cardiac Events, *UA* unstable angina, *RF* Ramadan Fasting, *Non-STEMI* Non-ST-Segment Elevation Myocardial Infarction, *TIA* Transient Ischemic Attack, *STEMI* ST-segment Elevation Myocardial Infarction, *TLR* Target Lesion Revascularization

In Group II (n = 150), we found eight MACEs during the follow-up. We admitted three patients with NSTEMI or UA (2.0%). We had two patients with STEMI; one patient had stent thrombosis while the other had a culprit lesion that was not related to the index PCI’s target lesion. We also admitted one patient with cerebrovascular stroke (0.66%). For the remaining two patients (1.3%), we admitted one of them with symptomatic hypotension because of severe gastroenteritis with severe dehydration. We admitted the other one with upper gastrointestinal bleeding (major bleeding event), as shown in Table 6.

**Table 6:** The relation between MACEs and the days from index PCI to the first day of Ramadan in Group II

	The days from index PCI to the first day of the following Ramadan in Group II (n = 150)													
	60–90		91–110		111–130		131–150		151–200		201–270		Total	
	N	%	N	%	N	%	N	%	N	%	N	%	N	%
Hospitalization for Non-STEMI or UA	1	0.66	1	0.66	0	0	1	0.66	0	0	0	0	3	2.6
STEMI	1	0	0	0	1	0.66	0	0	0	0	0	0	2	1.3
Death	0	0	0	0	0	0	0	0	0	0	0	0	0	0
Stroke or TIA	0	0	0	0	0	0	0	0	0	0	1	0.66	1	0.66
Hospitalization for other cardiovascular reasons	1	0.66	0	0	0	0	0	0	1	0.66	0	0	2	1.3
Total patients with MACEs	3	2	1	0.66	1	0.66	1	0.66	1	0.66	1	0.66	8	5.33

*MACEs* Major Adverse Cardiac Events, *UA* unstable angina, *RF* Ramadan Fasting, *Non-STEMI* Non-ST-Segment Elevation Myocardial Infarction, *TIA* Transient ischemic attack, *STEMI* ST-segment Elevation Myocardial Infarction

**Fig. 3:**
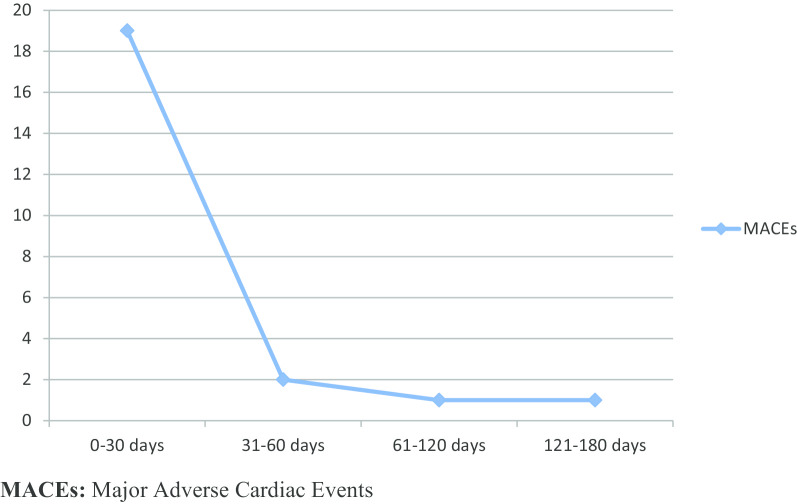
Distribution of MACEs during the follow-up period in Group I

We noticed that the incidence of MACEs in Group I was mainly during RF, with 19 patients (12.4%) having MACEs during this period and four patients (2.6%) during the remaining of the follow-up period. Besides, all the MACEs related to coronary thrombosis (n = 7) occurred during RF, as shown in Table 5 and Fig. 3.

**Fig. 4:**
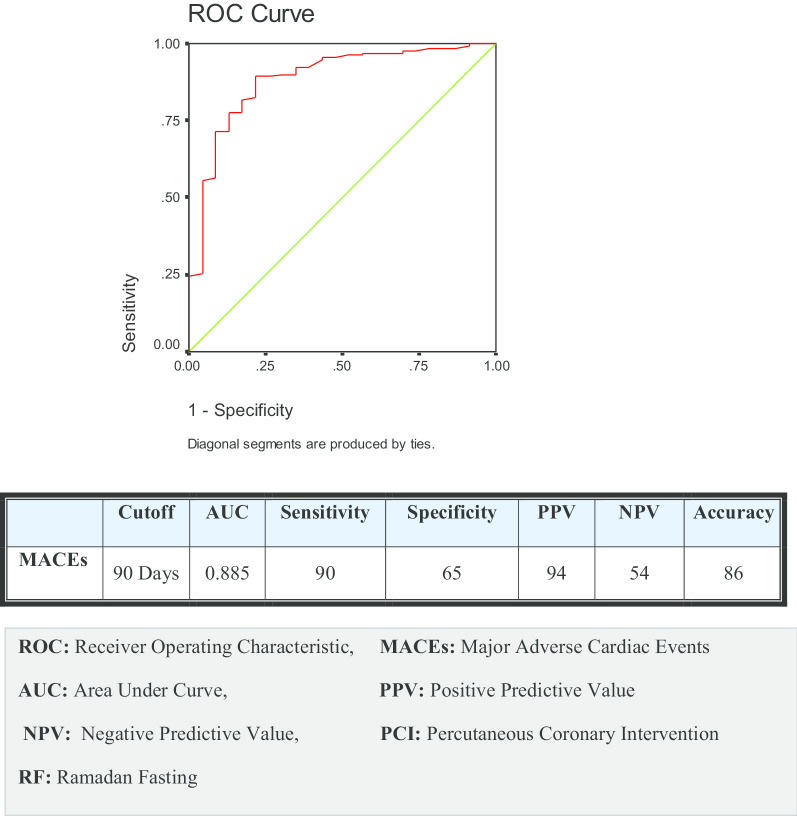
ROC curve for the relation between MACEs and the days from index PCI to the first day of RF in Group I

The univariate analysis for different predictors of MACEs in Group I showed that diabetes, duration from index PCI till the start of RF, SYNTAX score > 22, and complex procedure (bifurcation and multiple overlapped stents) were predictors of MACEs, with {*P* = 0.001, OR (5.113), *P* = 0.001, OR (1.523), *P* = 0.014 OR (3.020), and *P* = 0.001OR (3.013)}, respectively. We performed a multivariate logistic regression analysis to adjust the different potential confounders for MACEs, including {gender, age, risk factors (diabetes, hypertension, smoking, and dyslipidemia), and different aspects of PCI procedures (duration from index PCI till the start of RF, SYNTAX score > 22, DES type, and complex procedure)}. After adjustment of all the predictors, we found that duration from index PCI till the start of RF, SYNTAX score > 22, and complex procedure were independent predictors of MACEs in the fasting patients with {*P* = 0.001, OR (2.302), *P* = 0.026, OR (2.419), and *P* = 0.032 OR (1.952)}, respectively, as shown in Table 7. We performed a ROC curve analysis of our data and found the cutoff value at which the incidence of MACEs started to decline was 90 days from PCI till the start of RF with Sensitivity and specificity (90% and 65%), respectively, as shown in Fig. 4.

**Table 7:** Univariate and multivariate regression analysis of all the predictors of MACEs in Group I

	Univariate		Multivariate	
	Odds ratio (95% CI)	*P* value	Adjusted odds ratio (95% CI)	*P* value
Gender	1.352 (0.497–3.678)	0.821	1.458 (0.377–5.634)	0.585
DM	5.113 (1.940–13.479)	0.001*	2.027 (0.550–7.469)	0.288
HTN	0.754 (0.260–2.185)	0.523	0.426 (0.111–1.632)	0.213
Smoking	1.506 (0.586–3.867)	0.769	1.288 (0.339–4.890)	0.710
Dyslipidemia	1.231 (0.502–3.018)	0.912	1.090 (0.342–3.474)	0.884
Days from index PCI to the first day of RF	1.523 (1.105–5.308)	0.001*	2.302 (1.600–3.311)	0.001*
P2Y12 inhibitor (clopidogrel or ticagrelor)	0.881 (0.354–2.190)	0.802	0.819 (0.217–3.092)	0.768
PCI cause (elective or ACS)	2.302 (0.739–7.164)	0.523	1.956 (0.376–10.173)	0.425
SYNTAX score > 22	3.020 (1.220–7.574)	0.014*	2.419 (2.036–5.826)	0.026*
Stent type (Non-sirolimus eluting or sirolimus DES)	1.075 (0.335–3.450)	0.394	3.628 (0.831–9.324)	0.563
Complex procedure (bifurcation and multiple overlapped stents)	3.013 (1.117–8.127)	0.001*	1.952 (1.537–7.524)	0.032*

*CI* Co-Incidence Interval, *DM* diabetes, *HTN* hypertension, *PCI* Percutaneous Coronary Intervention, *MACEs* Major Adverse Cardiac Events, *RF* Ramadan Fasting

*Significant *P* value

## Discussion

Our study showed that the duration from index PCI till the start of RF, SYNTAX score > 22, and Complex procedure were independent predictors of MACEs. The decline of the incidence of MACEs occurred after 90 days from PCI till the start of RF with Sensitivity and specificity (90% and 65%), respectively.

Chamsi-Pasha and Ahmed found minimal clinical and biochemical effects of RF with heart disease. However, their study included a small number of patients with CAD, and only 11 patients underwent PCI [2]. Our study included a larger number of patients with CAD, and all the patients underwent relatively recent PCI. Also, we studied the control (non- fasting) group.

Al Suwaidi et al. [12] enrolled more than 1000 consecutive ACS patients admitted to the hospital in 9 months (including a Ramadan month) and found a lower relative risk for ACS in the fasting patients. The lower risk in the fasting group supports our finding that RF may be safe in patients with a history of ACS a few months after PCI. Our study defined ischemic patients’ stability after PCI when having a non-complex procedure and a syntax score ≤ 22. It also clarified a cut off value for the number of days from PCI to initiate relatively safe RF (> 90 days from index PCI).

Mousavi et al. studied 148 patients with documented CAD and normal EF. In their study, none of the patients with a history of revascularization reported chest pain during Ramadan [3]. In their research, only 66 intended to fast Ramadan, and 82 patients did not fast. They included fewer patients and did not highlight the duration between PCI to the initiation of RF as a possible predictor of chest pain recurrence. In our study, we studied the potential predictors of the MACEs, including the interval between PCI and the first day of RF, and we included the details of PCI procedure and patients' coronary anatomy complexity. In our study, we added a control group to confirm that our results were related to RF.

Nematy et al. included 82 patients with at least one cardiovascular risk factor, and they found significant improvement in 10 years of CAD risk (based on Framingham risk score) after RF. They explained this improvement by the higher High-Density Lipoprotein, and the lower Low-Density Lipoprotein, triglycerides, Very Low-density Lipoproteins, waist circumference, body mass index, and systolic blood pressure after RF [1]. This improvement after RF may explain our findings that RF may be safe after a few months of PCI, and it may be even safer in patients with lower risk profiles. Also, the relatively few MACEs in our study could be explained by the inhibited sympathetic tone during RF [13] and the limitation on smoking during Ramadan [14].

Bouida et al. found that RF was significantly associated with diminished platelet sensitivity to clopidogrel in diabetic patients during and shortly after Ramadan [15]. This potential adverse effect of RF was not clinically evident in our study, mostly because we excluded most diabetic patients (uncontrolled DM, IDDM, and those with significant renal impairment). Also, the degree of decreased platelet sensitivity to clopidogrel may not be sufficient to affect the clinical outcome.

Al Suwaidi et al. found that changing the drug timetable during the daylight due to fasting may affect cardiac patients [16]. In our study, the cornerstone of medications was the long-acting dual antiplatelet therapy. So, the effect of changing the drug schedule did not cause a significant unfavorable impact.

On the other hand, fasting inhibits catecholamine and reduces venous return. The catecholamine inhibition reduces the sympathetic tone, a fall in blood pressure, heart rate, and cardiac output [17]. On the other hand, this may lead to coronary slow flow and explain the increased incidence of MACEs in patients with recently deployed stent before RF.

Bener et al. confirmed that RF reduced blood pressure, HbA1C, and BMI. These beneficial effects extended 3 months after Ramadan [5]. On the other hand, Persynaki et al. found that RF improved body composition, lipidemia, and glycemia only during RF [18]. In our study, most of the MACEs occurred during RF due to thrombosis. Our findings may suggest that the only potential hazardous effect of early fasting after PCI may be related to dehydration and not due to any other metabolic disruption.

## Limitations of the study

Our study is not a randomized study because; we didn’t obligate the patients to fast. We included a few numbers of Primary PCI and ACS patients, and this may affect our findings regarding those important subgroups. We also excluded many diabetic patients (IDDM, renal impairment, and uncontrolled DM). Also, our study may not be illustrative because we conducted it in one country during the summertime, a period characterized by extended fasting hours and high temperatures. If RF was during winter, this might lead to fewer MACEs. So, we need to study this subject in different regions because the results may be affected by eating habits, the daylight hours, the climate, and the ability to reschedule the prescription. Also, the sample size was relatively small. Finally, we excluded the patients with MACEs that occurred before the first day of Ramadan. But, this did not affect our findings because we found no significant difference between the two groups regarding the days from index PCI to the first day of Ramadan.

## Conclusions

We suggest that RF during the hot and extended fasting hours in the summertime may not be safe during the first 3 months following the PCI in patients with high SYNTAX (score > 22) and who had a complex PCI procedure. We suggest that low-risk patients with a normal systolic function who underwent PCI may safely fast Ramadan. We could not apply this relative safety to the patients with any other comorbidity that may exempt them from RF for more prolonged periods after PCI. We recommend that this conclusion should be replicated in a larger multicenter study in different climate zones.

## Data Availability

The datasets used and or analyzed during the current study are available from the corresponding author on reasonable request.

## Abbreviations

RF: Ramadan Fasting
CAD: Coronary Artery Disease
PCI: Percutaneous Coronary Intervention
MACEs: Major Adverse Cardiac Events
EF: Ejection Fraction
IDDM: Insulin Dependent Diabetes Mellitus
DM: Diabetes Mellitus
NYHA: NEW YORK Heart Association functional class
NSTEMI: Non-ST Elevation Myocardial Infarction
UA: Unstable angina
STEMI: ST-Segment Elevation Myocardial Infarction
TIA: Transient Ischemic Attack
TLR: Target Lesion Revascularization
SPSS: Statistical Program for Social Science
SD: Standard Deviation
ROC: Receiver Operating Characteristic curve analysis
